# Atrial Fibrillation Complicated by Heart Failure Induces Distinct Remodeling of Calcium Cycling Proteins

**DOI:** 10.1371/journal.pone.0116395

**Published:** 2015-03-16

**Authors:** Patrick Lugenbiel, Fabian Wenz, Katharina Govorov, Patrick A. Schweizer, Hugo A. Katus, Dierk Thomas

**Affiliations:** Department of Cardiology, Medical University Hospital, Im Neuenheimer Feld 410, D-69120 Heidelberg, Germany; University of Canberra, AUSTRALIA

## Abstract

Atrial fibrillation (AF) and heart failure (HF) are two of the most common cardiovascular diseases. They often coexist and account for significant morbidity and mortality. Alterations in cellular Ca^2+^ homeostasis play a critical role in AF initiation and maintenance. This study was designed to specifically elucidate AF-associated remodeling of atrial Ca^2+^ cycling in the presence of mild HF. AF was induced in domestic pigs by atrial burst pacing. The animals underwent electrophysiologic and echocardiographic examinations. Ca^2+^ handling proteins were analyzed in right atrial tissue obtained from pigs with AF (day 7; n = 5) and compared to sinus rhythm (SR) controls (n = 5). During AF, animals exhibited reduction of left ventricular ejection fraction (from 73% to 58%) and prolonged atrial refractory periods. AF and HF were associated with suppression of protein kinase A (PKA)_RII_ (-62%) and Ca^2+^-calmodulin-dependent kinase II (CaMKII) δ by 37%, without changes in CaMKIIδ autophosphorylation. We further detected downregulation of L-type calcium channel (LTCC) subunit α_2_ (-75%), sarcoplasmic reticulum Ca^2+^-ATPase (Serca) 2a (-29%), phosphorylated phospholamban (Ser16, -92%; Thr17, -70%), and phospho-ryanodine receptor 2 (RyR2) (Ser2808, -62%). Na^+^-Ca^2+^ exchanger (NCX) levels were upregulated (+473%), whereas expression of Ser2814-phosphorylated RyR2 and LTCCα_1c_ subunits was not significantly altered. In conclusion, AF produced distinct arrhythmogenic remodeling of Ca^2+^ handling in the presence of tachycardia-induced mild HF that is different from AF without structural alterations. The changes may provide a starting point for personalized approaches to AF treatment.

## Introduction

Effective and safe management of atrial fibrillation (AF) constitutes a major clinical challenge. In particular, the co-existence of heart failure (HF), a common clinical scenario, is associated with increased morbidity and mortality, more rapid disease progression, and reduced efficacy of pharmacologic or interventional treatment of the arrhythmia [1–3]. Progressive electrical atrial remodeling of ion currents and calcium handling supports initiation and perpetuation of the rhythm disorder. The analysis of underlying mechanisms provides options for target-specific therapy that is required to improve outcome of AF patients [4–6]. However, mechanism-based approaches to AF therapy in cases with concomitant HF are currently limited by an insufficient understanding of molecular remodelling in HF-associated AF. Abnormalities in calcium cycling associated with either AF or HF have been studied previously. In AF, reduced L-type calcium channel (LTCC) current [7] may explain action potential shortening that was observed among chronic (but not paroxysmal) AF patients [8,9]. In addition, increased Ca^2+^-calmodulin-dependent kinase II (CaMKII) activity *via* autophosphorylation, ryanodine receptor type 2 (RyR2) hyperphosphorylation at Ser2808 and Ser2814, and Na+-Ca^2+^ exchanger 1 (NCX1) upregulation were implicated in delayed afterdepolarizations in chronic AF [8]. When analyzed in the absence of AF, atrial remodeling in canine HF is characterized by increased action potential duration (APD), decreased LTCC current, and enhanced NCX [10,11].

Ca^2+^-handling alterations that promote AF in HF subjects are poorly understood despite the high prevalence of AF in HF. Initial data obtained from dogs point towards a molecular interaction of AF and HF in phenotypic expression of critical components of cellular Ca^2+^ cycling that differs from the sum of effects observed in AF and HF alone, respectively [11]. The aim of the present study was to delineate specific molecular determinants of Ca^2+^ remodeling in AF and coexisting mild HF. An established porcine model of atrial tachypacing-induced AF complicated by HF [12–16] was employed to elucidate expression and phosphorylation status of Ca^2+^ cycling proteins in right atrial tissue samples.

## Materials and Methods

### Atrial fibrillation / heart failure animal model

AF was induced by rapid atrial burst pacing in sex-matched domestic swine (<6 months of age; body weight 25 to 30 kg). Animals were sedated with ketamine (100 mg/kg; Roche, Grenzach-Wyhlen, Germany) and midazolam (15 mg/kg, i.m.; Roche), and anesthetized with disoprivane (1 ml 1% solution; Astra Zaneca, Wedel, Germany) and isoflurane (1–2%; Baxter, Unterschleißheim, Germany). A pacing lead (4024 CapSure SP, Medtronic, Minneapolis, MN, USA) was inserted under fluoroscopic guidance through the right external jugular vein, fixed in the right atrial appendage, and connected to a pacemaker unit (Medtronic Kappa; Medtronic) placed in a subcutaneous pocket in the right neck. The pacemaker was programmed to apply burst pacing for the entire follow-up period at a frequency of 42 Hz for 2 seconds followed by 2 second intervals where the animals revealed their endogenous heart rate and rhythm. Under equivalent conditions pigs usually develop AF within 1–7 days [12–14,16]. During the 7 day observation period, daily 6-lead ECG recordings were performed to verify AF and to average ventricular response rates that were similar during pacing compared to non-paced intervals. ECG measurements were done while feeding in awake and alert animals. In addition, cardiac tissue samples were obtained from healthy domestic swine during sinus rhythm (SR) with similar weight and age that were not subjected to functional evaluation. This study has been carried out in accordance with the Guide for the Care and Use of Laboratory Animals as adopted and promulgated by the U.S. National Institutes of Health (NIH publication No. 86–23, revised 1985) and with EU Directive 2010/63/EU, and the current version of the German Law on the Protection of Animals was followed. The study has been approved by the Regierungspräsidium Karlsruhe (Karlsruhe, Germany; approval number G-106/10).

### Electrophysiological study

Electrophysiological (EP) examination was performed in AF animals during SR before pacemaker implantation and after electrical cardioversion before euthanization. Bipolar catheters were placed in the right atrium *via* the jugular vein. The EP Lab system (Bard Electrophysiology Division, Lowell, MA, USA) was used to record atrial effective refractory periods (AERP) and corrected sinus node recovery times (SNRT). To determine AERP, repeated trains of 10 stimuli at a fixed cycle length of 500 ms or 400 ms were applied, followed by a single programmed premature stimulus. The coupling interval between the last basic stimulus and the premature stimulus was decreased in 10 ms-steps until no atrial response was recorded. To assess corrected SNRT stimuli with fixed cycle lengths (500 ms and 400 ms) were applied for 30 seconds. The corrected SNRT was calculated by subtracting the intrinsic cycle length from the recorded recovery time.

### Echocardiographic examination

Measurements were carried out on the day of pacemaker implantation and at the end of the study (Philips Healthcare Sonos 5500, Hamburg, Germany) during SR following electrical cardioversion. Left ventricular ejection fraction (LVEF) was determined from M-mode measurements as described [14,17].

### Western blot analysis

Protein immunodetection was performed by sodium dodecyl sulfate (SDS) gel electrophoresis and Western blotting. The right atrial appendage was dissected, rapidly frozen in liquid nitrogen and stored at -80°C. Samples were homogenized (Yellow line DI 18 basic homogenizer, IKA, Essex, UK), followed by cell lysis in buffer containing 20 mM Tris-HCl, 0.5% NP-40, 0.5% sodium-deoxycholate, 150 mM NaCl, 1 mM EDTA, 1 mM Na_3_VO_4_, 1 mM NaF, and inhibitors of proteases (Complete) and phosphatases (PhosStop) (Roche Applied Science, Indianapolis, IN, USA). For preparation of sarcoplasmic reticulum fractions atrial tissues were homogenized on ice in a solution containing 0.3 M sucrose, 10 nM imidazole, 30 mM histidine, protease and phosphatase inhibitor (Mini Complete, PhosStop; Roche Applied Science, Indianapolis, IN, USA). Homogenates were centrifuged at 4°C for 20 min at 8000g. The supernatants were collected and centrifuged at 45,000g for 90 min. The pellets were resuspended in a solution containing 0.3 M sucrose, 10 nM imidazole, 30 mM histidine and 0.6 M KCl, and centrifuged at 2000g to remove myofibrillar proteins. Finally, the supernatant was centrifuged at 45,000g for 90 min, and resulting pellets were resuspended in the solution described above.

The protein concentration was determined using the bicinchoninic acid (BCA) protein assay (Thermo Scientific, Rockford, IL, USA). Equal amounts of protein were separated on 6–20% SDS polyacrylamide gels, transferred to polyvinylidene difluoride membranes, and developed using primary antibodies directed against L-type calcium channel (LTCC) α_1c_ subunit (sc-25686; Santa Cruz Biotechnology, Heidelberg, Germany), LTCC α_2_ subunit (ab62814; Abcam), Na^+^-Ca^2+^ exchanger (NCX) 1 (ab2869; Abcam), catalytic protein kinase A (PKA) subunits Cα/β (ab26322; Abcam), phosphorylated regulatory PKA subunit RIIα (Ser99) (ab32390; Abcam), phospholamban (PLN) (MA3-922; Thermo Scientific, Dreieich, Germany), phosphorylated PLN (Ser16) (07-052; Upstate (Merck Millipore), Billerica, MA, USA), phosphorylated PLN (Thr17) (sc-17024-R; Santa Cruz Biotechnology, Heidelberg, Germany), Ca^2+^-calmodulin-dependent protein kinase II (CaMKII) δ (C1035-04E; Biomol, Hamburg, Germany), autophosphorylated CaMKIIδ (Thr286) (V1111; Promega, Madison, WI, USA), ryanodine receptor type 2 (RyR2) (ab2868; Abcam), phosphorylated RyR2 (Ser2808) (A010-30; Badrilla, Leeds, UK), phosphorylated RyR2 (Ser2814) (A010-31AP; Badrilla), and sarcoplasmic reticulum Ca^2+^-ATPase (Serca) 2a (sc-8095; Santa Cruz Biotechnology). Horseradish peroxidase (HRP)-conjugated goat anti-rabbit (ab6721; Abcam), mouse anti-goat (sc-2354; Santa Cruz Biotechnology), and goat anti-mouse (sc-2005; Santa Cruz Biotechnology) secondary antibodies were used where appropriate.

Signals were developed using the enhanced chemiluminescence assay (ECL Western Blotting Reagents, GE Healthcare, Buckinghamshire, UK) and quantified with ImageJ 1.41 Software (National Institutes of Health, Bethesda, MD, USA). After removal of primary and secondary antibodies, the membranes were reprobed with anti-glyceraldehyde 3-phosphate dehydrogenase (GAPDH) antibodies (G8140-11; US Biological, Swampscott, MA, USA) and corresponding secondary antibodies (sc-2005; Santa Cruz Biotechnology). Protein content was normalized to GAPDH for quantification of optical density.

### Statistics

Data are expressed as mean ± SEM of n experiments. We used paired and unpaired Student’s *t* tests (two-tailed tests) to compare the statistical significance of the results. *P* < 0.05 was considered statistically significant

## Results

### Atrial tachypacing-induced AF is associated with rapid ventricular response and impaired left ventricular function

**Fig 1:**
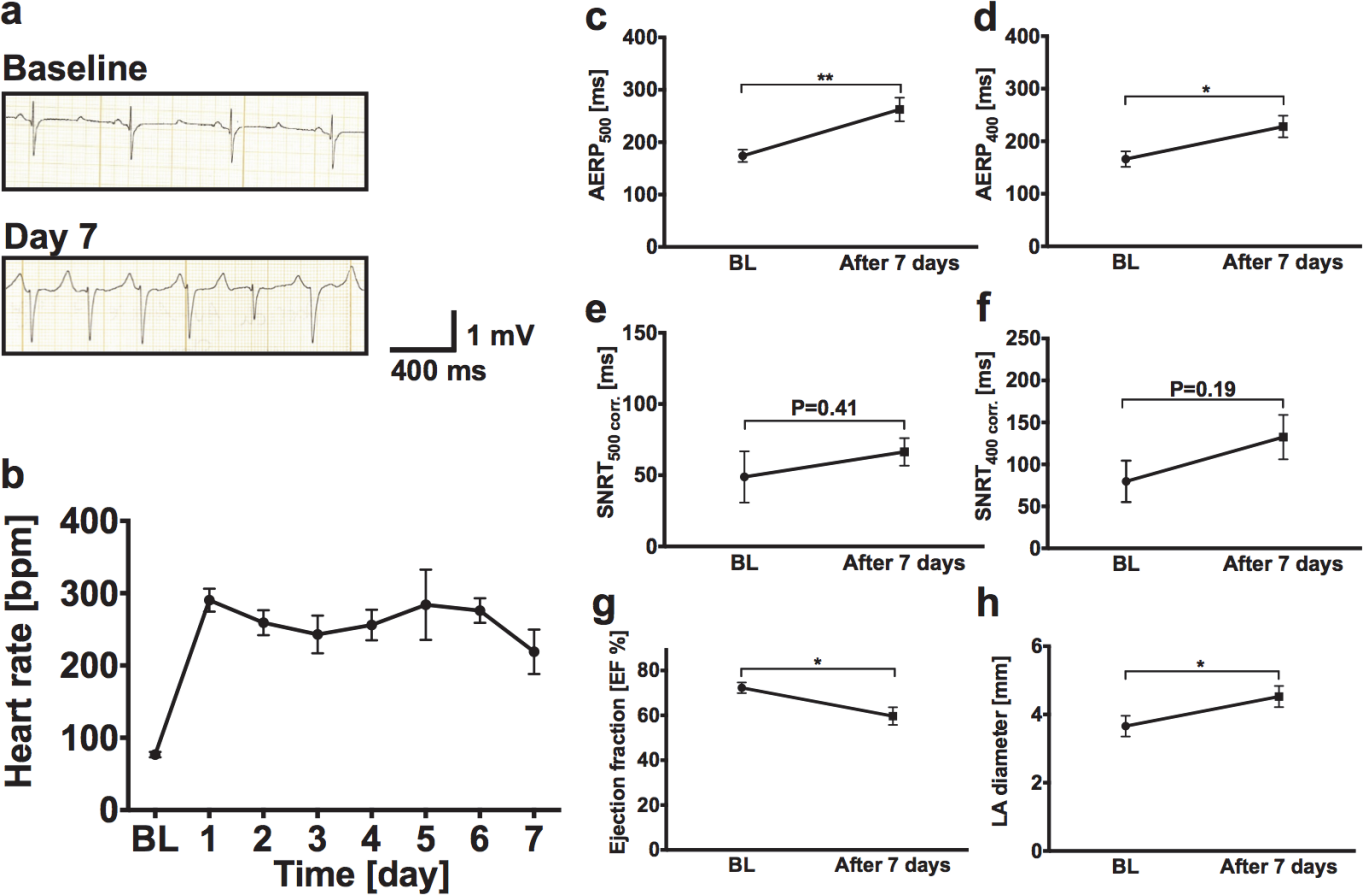
Clinical findings in AF pigs subjected to atrial tachypacing. **a** Representative ECG recordings at baseline and after atrial fibrillation (AF) induction by atrial burst pacing show sinus rhythm on day 0 and AF on day 7. **b** Mean heart rates assessed by daily ECG measurements from AF animals (n = 5). **c-f** Atrial effective refractory periods (AERP; **c**, **d**) and corrected sinus node recovery time (SNRT; **e**, **f**) at baseline and prior to euthanization at day 7 in AF pigs (n = 5). **g, h** Echocardiographic analysis of left ventricular ejection fraction (EF) and left atrial (LA) diameter. Data are given as mean ± SEM; *P < 0.05; **P < 0.01.

Atrial tachypacing successfully induced AF in all animals following pacemaker implantation (Fig. 1A). AF was characterized by rapid ventricular response rates during follow-up (mean heart rate, 249 ± 49 beats per minute (bpm); n = 5; Fig. 1B). Electrophysiological examinations were performed prior to pacemaker implantation and at the end of the 7 day period to determine AERP. We observed AERP prolongation at cycle lengths of 500 ms (baseline, AERP_500_ = 174 ± 26 ms; day 7, 263 ± 45 ms; n = 5; *P* = 0.007; Fig. 1C) and 400 ms (baseline, AERP_400_ 166 ± 33 ms; day 7, 228 ± 46 ms; n = 5; *P* = 0.040; Fig. 1D). Corrected SNRT under baseline conditions and at the end of the study were not significantly different at 500 ms basic cycle length (BCL) (49 ms ± 40 ms at baseline *versus* 66 ms ± 22 ms on day 7; n = 5; *P* = 0.41) and at 400 ms BCL (80 ms ± 49 ms at baseline *versus* 133 ms ± 53 ms on day 7; n = 5; *P* = 0.19), respectively (Fig. 1E and F).

Uncontrolled tachycardia during AF resulted in impairment of left ventricular ejection fraction (LVEF). Echocardiograms performed at the beginning of the study during sinus rhythm revealed normal function (LVEF = 73 ± 6%; n = 5). In contrast, LVEF was significantly reduced to 58 ± 8% (n = 5; *P* = 0.033) at sacrifice (day 7; Fig. 1G). Furthermore, the mean LA diameter increased from 34 ± 7 mm prior to initiation of the burst pacing program to 44 ± 7 mm after 7 days (n = 5; *P* = 0.013; Fig. 1H).

### Alterations of protein kinase expression during AF and HF

**Fig 2:**
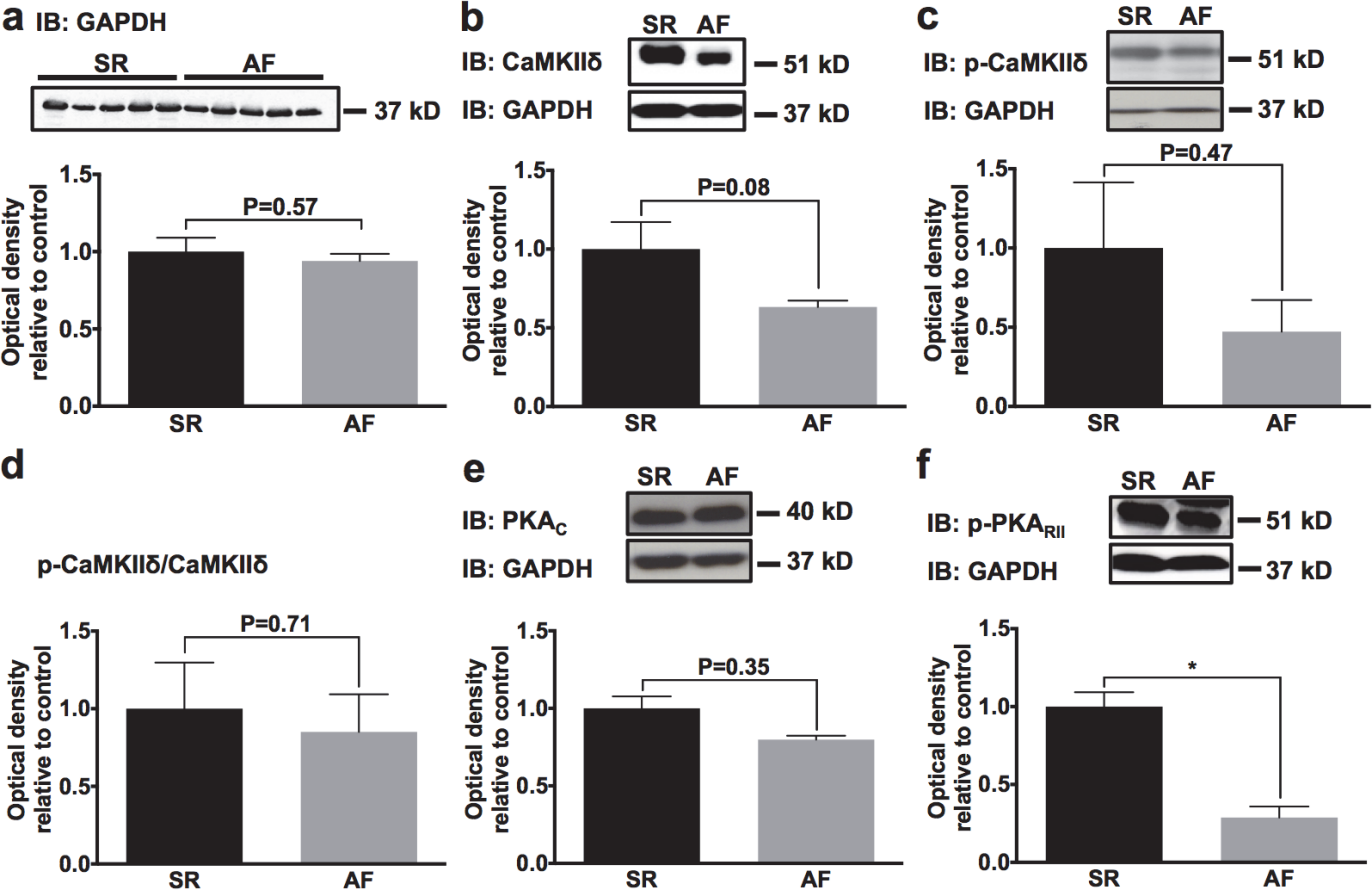
AF-induced remodeling of Ca^2+^-calmodulin-dependent protein kinase II (CaMKII) δ and protein kinase A (PKA). Representative Western blots and mean optical density values normalized to glyceraldehyde 3-phosphate dehydrogenase (GAPDH) are displayed relative to sinus rhythm (SR) controls. **a,** Absolute expression levels of the reference protein, GAPDH, were note affected by AF. **b, c** Normalized protein expression of total CaMKIIδ and phosphorylated CaMKIIδ (Thr286) in AF animals compared to SR. **d** Relative CaMKIIδ autophosphorylation at Thr286 was not different between animal groups. **e, f** Protein levels of catalytic PKA Cα/β subunits (PKA_C_) and of phosphorylated regulatory RIIα subunits (PKA_RII_) in AF and SR animals. Mean values obtained from five animals per group are provided ± SEM; *P < 0.05.

To delineate remodeling of Ca^2+^-handling during AF in the presence of mild HF, Western blot analyses were performed in five pigs subjected to atrial tachypacing for 7 days. Pigs exhibiting SR that were not subjected to AF induction served as controls (n = 5). We first assessed alterations of key upstream kinases that are responsible for functional regulation of Ca^2+^ cycling proteins through reversible phosphorylation, PKA and CaMKIIδ. The reference protein, GAPDH, did not exhibit AF-associated remodeling (Fig. 2A) in accordance with previous studies [18]. AF was associated with 37% decrease of CaMKIIδ expression compared to SR controls (*P* = 0.08; Fig. 2B). There was no significant change of CaMKIIδ autophosphorylation (i.e. persistent activation) at Thr286 in AF animals (*P* = 0.47; Fig. 2C). Relative levels of autophosphorylated / total CaMKIIδ remained unchanged as well (Fig. 2D). Downregulation of PKA-phosphorylated regulatory PKA RIIα subunits (at Ser99) by 62% (*P* = 0.017) indirectly reflected reduced activity of the catalytic PKA chain during AF. Total catalytic PKA Cα/β levels were similar between AF and SR animals (*P* = 0.35) (Fig. 2E and F).

### Remodeling of Ca^2+^- handling protein expression and phosphorylation in AF / HF pigs

**Fig 3:**
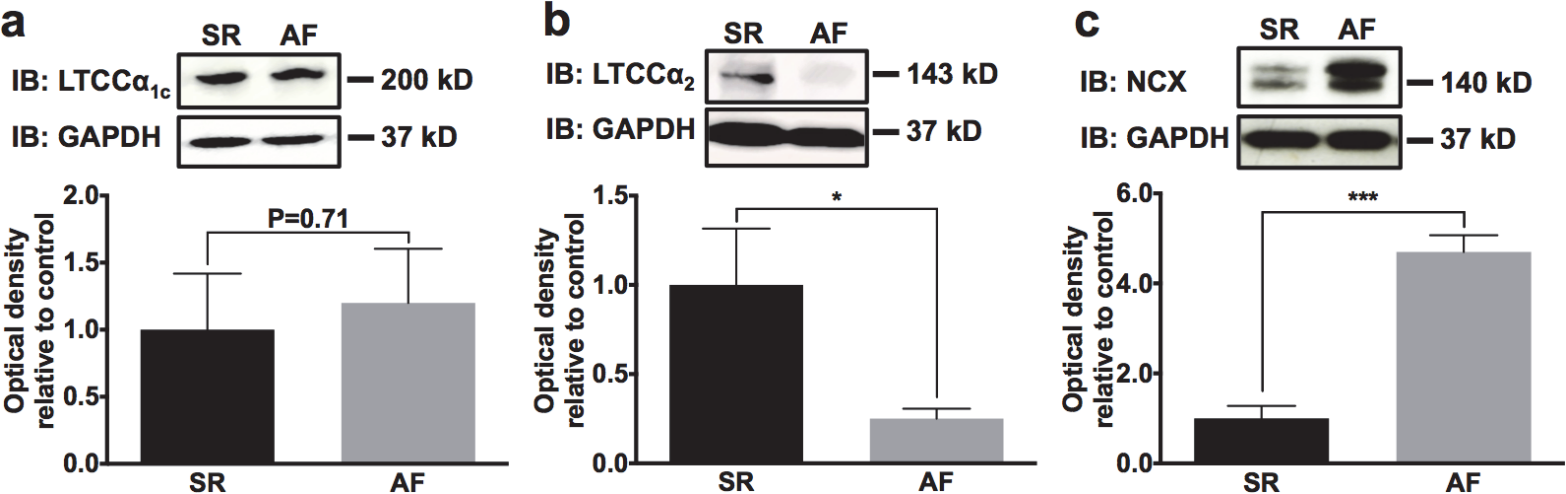
Expression of L-type Ca^2+^ channels (LTCC) and Na+-Ca^2+^ exchanger (NCX) 1 in AF animals and in SR controls. **a, b** Regulatory LTCC α_2_ subunit was significantly downregulated in AF animals, whereas the pore forming α_1c_ subunit was not affected. **c** AF lead to significant upregulation of the NCX1 transporter. Representative Western blots and mean (± SEM) optical density data normalized to GAPDH are displayed (n = 5 animals per group). *P < 0.05; ***P < 0.001.

**Fig 4:**
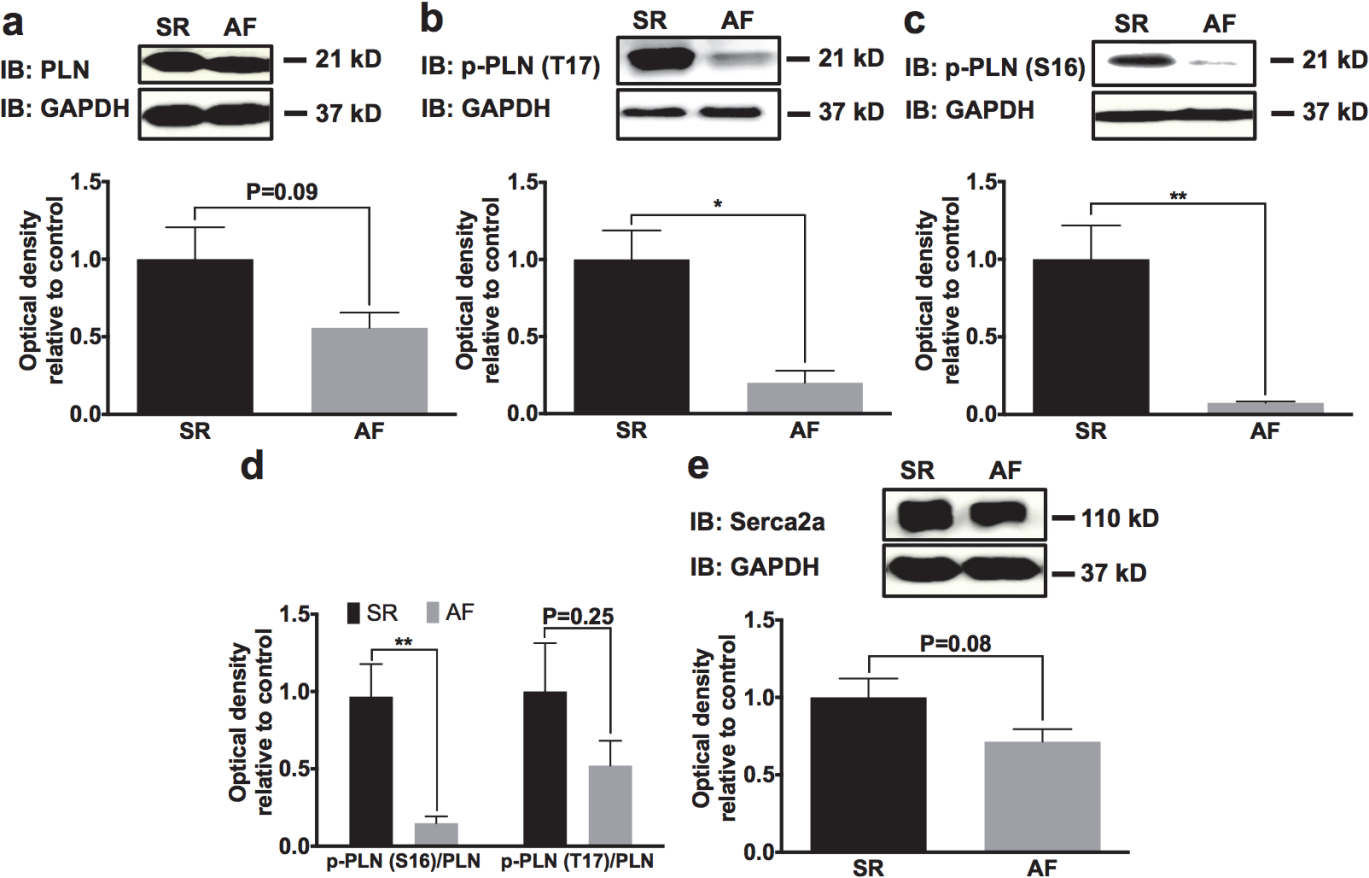
Alterations of sarcoplasmic reticulum Ca^2+^-ATPase (Serca) 2a and its regulator phospholamban (PLN) during AF. Total PLN (**a**) and PLN phosphorylated by CaMKII (Thr17; **b**) or PKA (Ser16; **c**) was downregulated in AF animals. **d** Phosphorylation levels relative to total PLN. **e** Serca2a protein analysis revealed a trend towards reduced protein expression associated with AF. Mean ± SEM optical density data normalized to GAPDH obtained from n = 5 Western blots per group are displayed. *P < 0.05; **P < 0.01.

To further assess alterations of Ca^2+^-signaling during AF complicated by functional LV impairment, we investigated expression of L-type calcium channel α subunits. LTCC α_2_ subunit levels were significantly lower in AF animals compared to SR (-75%; n = 5; *P* = 0.047), whereas the α_1c_ subunit was not affected by atrial burst pacing-induced AF and HF (n = 5; *P* = 0.71) (Fig. 3A and B). In contrast to LTCC downregulation, NCX1 expression was increased in animals with AF (+473%; n = 5; *P* < 0.0001; Fig. 3C). In addition to LTCC and NCX1 located in the cell membrane, the intracellular Ca^2+^ content is regulated by the Ca^2+^ pump Serca2a in the sarcoplasmic reticulum membrane and its endogenous inhibitor, PLN. After 7 days of AF with rapid response rate, total PLN levels showed a tendency towards downregulation that was not statistically significant compared to SR animals (n = 5; *P* = 0.09; Fig. 4A). Phosphorylation of PLN by CaMKII at Thr17 (-70%; n = 5; *P* = 0.014) and by PKA at Ser16 (-92%; n = 5; *P* = 0.003) was significantly reduced (Fig. 4B-D), indicating enhanced inhibitory effects of dephosphorylated PLN and diminished Serca2a activity. The effector pump, Serca2a, exhibited 29% reduction in AF *versus* SR that did not reach statistical significance (n = 5; *P* = 0.08; Fig. 4E).

**Fig 5:**
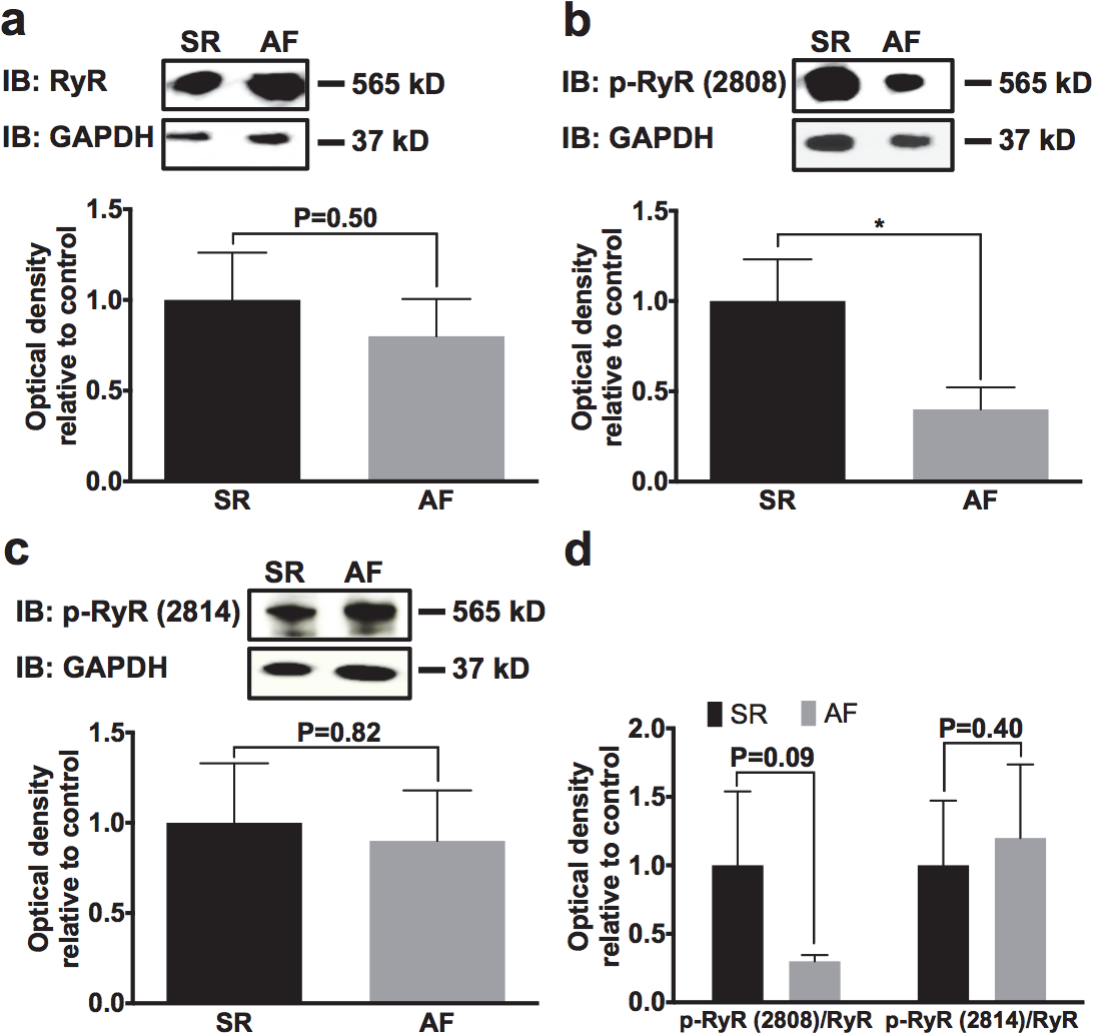
Analysis of ryanodine receptor (RyR) and phosphorylated ryanodine receptor (p-RyR) levels in SR and AF pigs. **a** Total RyR protein did not differ between animal groups. **b, c** PKA-phosphorylated RyR at Ser2808 was reduced, while CaMKII-phosphorylated RyR at Ser2814 was not affected by AF. **d** Relative p-RyR_Ser2808_ but not p-RyR_Ser2814_ content was diminished in atrial tissue obtained from n = 5 animals per group. Original Western blots and mean normalized optical density data are provided (*P < 0.05).

Ca^2+^ release from the sarcoplasmic reticulum is mediated through RyR2 channels. During AF with reduced LVEF in pigs, phosphorylation of RyR2 by PKA at Ser2808 was significantly reduced by 62% (n = 5; *P* = 0.040), whereas total RyR2 protein (n = 5; *P* = 0.50) and CaMKIIδ phosphorylation at Ser2814 (n = 5; *P* = 0.82) was not different between AF and SR animal groups (Fig. 5A-D).

## Discussion

### Remodeling of Ca^2+^ signaling in AF complicated by tachymyopathy: implications for cellular arrhythmogenesis

**Fig 6:**
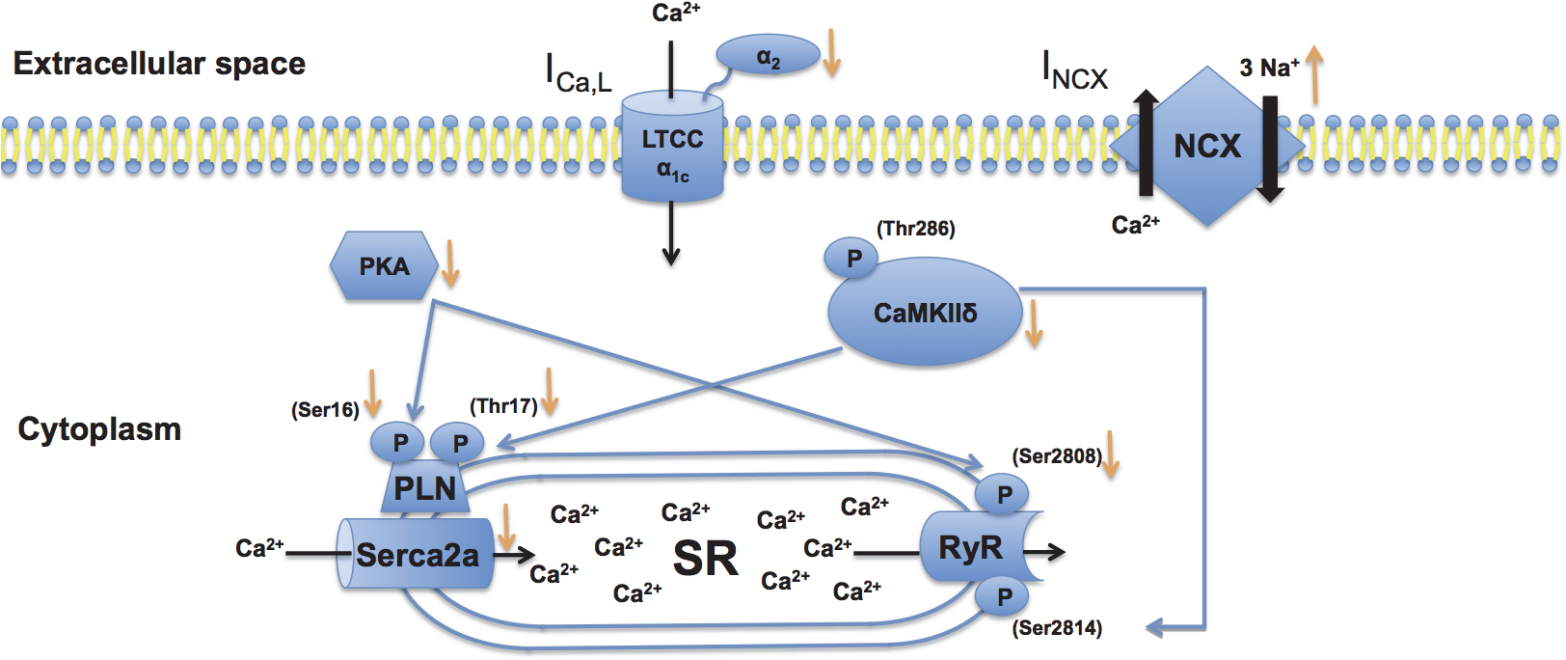
Remodeling of Ca^2+^ handling proteins during atrial fibrillation and heart failure. Changes in protein expression are indicated by arrows (orange color). Solid black arrows indicate transport directions of Ca^2+^ or Na^+^ ions, respectively. Decreased levels of Ca^2+^-calmodulin-dependent protein kinase (CaMK) IIδ and protein kinase A (PKA) causes hypophosphorylation of phospholamban (PLN) and ryanodine receptor (RyR) 2, leading to reduced Ca^2+^ uptake into the sarcoplasmic reticulum through sarcoplasmic reticulum Ca^2+^-ATPase (Serca) 2a. Increased intracellular Ca^2+^ levels and upregulation of Na^+^-Ca^2+^ exchanger (NCX) 1 expression enhance electrogenic Na^+^-Ca^2+^ exchange. This mechanism is attenuated by reduced L-type calcium channel (LTCC) expression that limits systolic Ca^2+^ influx.

This work reveals distinct alterations of Ca^2+^ signaling in a large animal model of AF and functional alterations of the LV. Here, AF and mild HF resulted in prolongation of AERP. This unexpected finding is attributed to reduced K^+^ channel expression [18,19] and highlights the distinction of this sub-entity of AF. Prolongation of atrial refractoriness in isolation will enhance LTCC-mediated depolarizing Ca^2+^ influx during the plateau phase of each action potential, leading to early afterdepolarizations (EADs). However, autoprotective LTCC downregulation (Fig. 6) directly limits Ca^2+^ entry to reduce toxic cytosolic Ca^2+^ overload, excessive ERP prolongation, and EAD generation. AF animals with diminished cardiac function exhibited decreased Serca2a expression (Fig. 6). In addition, dephosphorylated PLN that inhibits Serca2a function was associated with reduced expression of CaMKIIδ and phosphorylated regulatory RIIα PKA subunits after 7 days of AF. Functional cellular consequences of Ca^2+^ handling alterations and their impact on atrial arrhythmogenesis require further investigation.

### Comparison with previous studies in animal models and humans

Reduction of LVEF and enlarged left atrial diameters are consistent with tachymyopathy in predisposed human AF subjects. In most studies of human chronic (but not paroxysmal) AF [8,9] and in AF models that do not develop HF [5,11,20–23] action potential shortening has been reported. Thus, prolongation of atrial refractoriness observed in AF / HF pigs is a remarkable finding that may represent a hallmark of AF in combination with HF and reduced ejection fraction [24–27]. In humans, clinically and mechanistically relevant overlap between sinoatrial node (SAN) dysfunction and atrial fibrillation has been observed [28,29]. In dogs, AF induced by long term atrial burst pacing resulted in SAN dysfunction with prolonged SNRT [30,31]. We noted a trend towards prolonged SNRT in pigs after short term follow-up that may indicate AF-related impairment of SAN function in line with previous reports.

At the molecular level, proarrhythmic remodeling of Ca^2+^ handling was complex and exhibited specific characteristics that differed from findings obtained during AF or HF in isolation, respectively. Previous studies revealed that in the absence of relevant ventricular dysfunction, AF is primarily characterized by elevated NCX1 levels and LTCC downregulation [7,27,32]. Active PKA and CamKII are upregulated, leading to RyR2 phosphorylation at Ser2808 (PKA) and Ser2814 (CaMKII; observed in cAF but not pAF) that causes diastolic Ca^2+^ leak, DADs, and ectopic activity [8,9,23,27,32]. In addition, increased activity of regulatory protein kinases accounts for PLN phosphorylation. Phosphorylation relieves PLN inhibition of Serca2a and results in increased sarcoplasmic reticulum Ca^2+^ load that further supports DAD generation [23]. Serca2a protein levels are reduced in isolated AF [32]. Of note, complex and differing data on Ca^2+^ remodeling suggested that precise mechanisms likely differ between patient sub-populations [33]. Similar to AF in the absence of reduced LV function, the combined phenotype of AF and HF studied here displayed increased NCX1 and decreased LTCC and Serca2a levels, respectively (Fig. 6). In the present work, however, atrial remodeling of AF / HF animals was characterized by downregulation of phosphorylated PKA RIIα and CaMKII with subsequent hypophosphorylation of PLN and RyR2 (Fig. 6) in contrast to alterations in AF without relevant structural impairment of the heart.

In animal models or patients with HF, the downregulation of Serca2a and LTCC as well as NCX1 upregulation are consistent findings in atrial myocytes [6,7,32], similar to AF and AF / HF subjects. In addition, previous studies of atrial remodeling in HF revealed characteristic downregulation and hypophosphorylation of RyR2 [27,32]. The latter observation is shared by AF / HF pigs and distinguishes this disease sub-entity from “common” AF without reduced LV function. The primary mechanism of atrial arrhythmogenesis in HF appears to be increased CaMKII function and CaMKII-dependent PLN hyperphosphorylation that attenuate Serca2a inhibition, leading to Ca^2+^ overload of the sarcoplasmatic reticulum and DADs [27,32,34]. By contrast, the absence of these CaMKII-dependent changes appears to be unique to AF / HF hearts and has not been reported previously. Whether CaMKII downregulation and reduced phosphorylation of downstream proteins are specific characteristics of the porcine AF model or reflections of a general mechanism in an underexplored AF / HF sub-entity of the arrhythmia remains to be determined.

### Therapeutic implications

Distinct remodeling of Ca^2+^ cycling in AF / HF subjects requires specific therapeutic approaches. Class III antiarrhythmic drugs that further prolong action potential duration and AERP may be less effective in these cases. Decreased Serca2a expression and function provide potential targets for antiarrhythmic therapy. Therapeutic stimulation of Serca2a could reduce cytosolic Ca^2+^ levels and attenuate arrhythmogenic NCX1-mediated Ca^2+^ eliminiation. This would, however, be associated with sarcoplasmic reticulum Ca^2+^ overload, increasing the risk of diastolic Ca^2+^ leak and DADs. In addition, inhibition of L-type Ca^2+^ channels that are downregulated in AF / HF animals may protect from EAD generation and ERP prolongation, but negative inotropic effects render this concept unsuitable in HF patients. Finally, attenuation of AERP prolongation without negative effects on intracellular Ca^2+^ homoestasis could be achieved by specific activation of atrial-selective K^+^ channels (e.g. K_2P_ channels; [18,19]).

### Potential limitations and future directions

Experimental large animal models do not show spontaneous AF occurrence that is observed in AF patients. Nonetheless, the unique porcine model used in this work is comparable to the clinical scenario of AF and left ventricular dysfunction due to rapid ventricular rate response. Thus, we suggest that the pig is a valid model system to analyse atrial remodeling in the context of AF complicated by HF. Functional analyses were limited to *in vivo* studies among AF animals, precluding experimental confirmation of proposed arrhythmogenic mechanisms at the cellular level. The 7 day follow-up period in the present study reflects short-term remodeling processes during AF in subjects with slightly diminished ventricular function. For further evaluation of atrial Ca^2+^ cycling alterations, long term investigations of AF pigs with more advanced HF should be performed. Additional studies are required in AF patients with concomitant HF to further validate our data, as remodeling in experimental atrial tachypacing-induced AF with acute HF may differ from clinical AF / HF findings. This is particularly important because time-dependent effects during AF and chronic heart disease cannot be assessed during short-term studies in pigs. Finally, atrial-selective K_2P_ channel activation requires *in vitro* and *in vivo* evaluation as antiarrhythmic concept to treat AF in combination with HF.

## Conclusions

We present new insights into atrial remodeling of Ca^2+^ signaling in AF complicated by reduced ventricular function. Complex remodeling of proteins involved in Ca^2+^ cycling in AF / HF animals yields a distinct expression and phosphorylation pattern that cannot be explained by the summation of changes observed in isolated HF or AF. The data provide an explanation why the arrhythmia is particularly difficult to treat in a sub-entity of AF patients with concomitant HF, serving as a starting point for more personalized AF treatment.
